# Size-controllable synthesis of NiCoSe_2_ microspheres as a counter electrode for dye-sensitized solar cells

**DOI:** 10.1039/c8ra04091e

**Published:** 2018-07-19

**Authors:** Xiaobo Chen, Jingguo Ding, Yan Li, Yinxia Wu, Guoce Zhuang, Cuicui Zhang, Zhihai Zhang, Chengyun Zhu, Peizhi Yang

**Affiliations:** School of New Energy and Electronic Engineering, Yancheng Teachers University Yancheng 224051 PR China chenxbok@126.com; Key Laboratory of Education Ministry for Advance Technique and Preparation of Renewable Energy Materials, Institute of Solar Energy, Yunnan Normal University Kunming 650500 PR China pzhyang@hotmail.com

## Abstract

NiCoSe_2_ microspheres have been successfully synthesized by a facile one-step hydrothermal method at different hydrothermal temperatures. The prepared samples are divided according to their reaction temperatures (90, 120, 150 and 180 °C) and named NiCoSe_2_-90, NiCoSe_2_-120, NiCoSe_2_-150 and NiCoSe_2_-180, respectively. The diameters of the NiCoSe_2_ microspheres strongly depend on the different hydrothermal temperatures. When the temperature is increased to 150 °C, the size of the resultant NiCoSe_2_ microspheres changes from 200 to 800 nm, and the interior of NiCoSe_2_-150 possesses a flocculent structure. However, NiCoSe_2_-180 displays a cauliflower-like aggregated structure. The prepared NiCoSe_2_ alloys are used as high-performance Pt-free counter electrodes (CEs) for dye-sensitized solar cells (DSSCs). Cyclic voltammogram measurement indicates that NiCoSe_2_-150 CE has larger current density than Pt CE. Electrochemical impedance spectroscopy shows that NiCoSe_2_-150 CE has a low charge-transfer resistance of 1.8 Ω cm^2^. Due to their unique morphologies and well-defined interior and exterior structures, DSSCs based on NiCoSe_2_-120 and NiCoSe_2_-150 CEs achieve high power conversion efficiencies of 8.48% and 8.76%, respectively, which are higher than that of the solar cell based on Pt CE (8.31%).

## Abstract 2

The PCE value of the NiCoSe_2_-150-based DSSC is 8.76%, which is higher than that of DSSCs based on Pt (8.22%).

## Graphical abstract

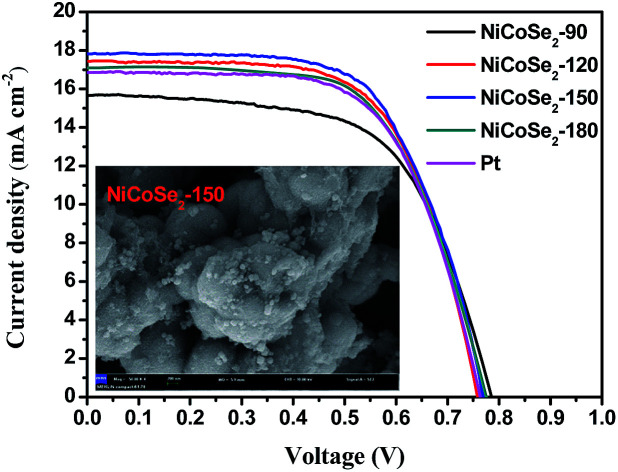

## 1. Introduction

Dye-sensitized solar cells (DSSCs) are regarded as a new type of promising solar cells because of their environmental friendliness, low cost, easy preparation processes and good photovoltaic performance.^1–3^ A typical DSSC usually consists of a counter electrode (CE), a dye-loaded TiO_2_ photoanode and a triiodide/iodide (I^−^/I_3_^−^) redox electrolyte. The main role of CE is to collect the electrons from the external circuit and return them back to the redox electrolyte and catalyze the reduction of I_3_^−^ to I^−^ at the CE/electrolyte interface.^4,5^ Pt-coated fluorine-doped tin oxide (FTO) glass is often used as CE in a general DSSC, which shows high performance due to its excellent conductivity, outstanding electrocatalytic activity and good chemical stability. However, until now, the most limiting factors in the development of commercial DSSCs have been their cost and long-term stability. The large-scale application of Pt CE in commercial DSSCs is limited, which has greatly stimulated the research of stable and effective Pt-free CE materials with low cost, high conductivity and electrocatalytic activity for the reduction of I_3_^−^.^6,7^

Several low-cost materials with excellent performances, such as alloys,^8,9^ transition metal compounds,^4,10,11^ carbonaceous materials,^12,13^ composites,^14,15^ and conductive polymers,^16,17^ have been successfully utilized as Pt-free CEs in DSSCs. Among these Pt-free CE materials, metal selenides are competitive candidates because of their outstanding electrocatalytic activity for triiodide reduction.^4,18–20^ A hollow NiSe–Ni_3_Se_2_ hybrid nanostructure composite was also directly synthesized on reduced graphene oxide *in situ*; the NiSe series exhibited a high PCE value of 7.87%.^21^ Profiting from the coexistence of Ni and Co in Ni–Co selenides, ternary selenides offer richer redox reactions than NiSe and CoSe. For example, (Ni_1−*x*_Co_*x*_)Se_2_ nanoparticles were synthesized *via* a one-step hydrothermal reduction route, and the obtained sample could exhibit better electrocatalytic properties compared to NiSe_2_, CoSe_2_ and Pt.^22^ Moreover, it is generally acknowledged that stoichiometric ratio and morphology are the two main factors that affect the catalytic activity of CE materials.

Herein, NiCoSe_2_ microspheres were prepared by a one-step facile hydrothermal method at different hydrothermal temperatures, and they were used as CE materials for DSSCs. The size of the microspheres increased with the increase in the reaction temperature, and the microspheres exhibited agglomeration. Meanwhile, the NiCoSe_2_-150 sample exhibited the most porous features. The overall energy conversion efficiency of the DSSC with NiCoSe_2_-150 CE reached 8.76%, and DSSCs with NiCoSe_2_-120 and NiCoSe_2_-180 CEs achieved power conversion efficiencies (PCE) of 8.48% and 8.31%, respectively. PCEs of the cells based on the above-mentioned three NiCoSe_2_ CEs were superior to that of Pt CE-based DSSC (8.22%).

## 2. Experimental

### 2.1. Preparation of NiCoSe_2_ microspheres

NiCoSe_2_ microspheres were synthesized *via* a facile one-step hydrothermal method. First, 1 mol of NiCl_2_·6H_2_O, 1 mol of CoCl_2_·6H_2_O and 0.2 mol of cetyl trimethylammonium bromide (CTAB) were mixed in 30 mL of distilled water. To this solution, 2 mol of Se powder was added and magnetically stirred for 15 min; 20 mL of N_2_H_4_·H_2_O was then added drop-wise to the reaction mixture on vigorous stirring for 30 min. After ultrasonic oscillation for 20 min, the resulting mixture was transferred to a Teflon-lined stainless-steel autoclave. The hydrothermal temperatures were set at 90, 120, 150 and 180 °C for 24 h. After cooling to room temperature, the product was centrifuged, cleaned repeatedly with distilled water and absolute ethanol to remove residual chemicals and then dried in a vacuum oven at 80 °C for 6 h. The obtained products were labeled as NiCoSe_2_-90, NiCoSe_2_-120, NiCoSe_2_-150 and NiCoSe_2_-180.

### 2.2. Preparation of counter electrodes

The counter electrodes of NiCoSe_2_ with different reaction temperatures were spread on cleaned FTO glass by spin-coating. First, 5 mg of the corresponding NiCoSe_2_ powder was put into 0.5 mL of absolute alcohol; after ultrasonic stirring for 30 min, a well-dispersed solution was formed. Then, the resulting NiCoSe_2_ dispersion solution was coated on the cleaned FTO glass by spin-coating with a rotation rate of 500 rpm for 15 s. After drying at 80 °C for 5 min, a second layer of NiCoSe_2_ was subsequently coated over the first layer. Then, a third layer was also deposited on the previous layer in the same way. After the deposition of the three layers, the coated FTO glass was dried at 140 °C for 15 min. The thickness of each electrode on the FTO glass was about 4 μm. A commercial Pt electrode (Dalian HepatChroma Solar Tech. Co., Ltd.) was used as a reference.

### 2.3. Fabrication of DSSCs

TiO_2_ photoanodes were prepared on an FTO glass substrate (7 Ω per square) by using the conventional screen-printing method.^23^ A TiO_2_ film (∼12 μm) was coated on the FTO glass as a transparent nanocrystalline-sensitized TiO_2_ layer using commercial 20 nm TiO_2_ pastes (Dalian HepatChroma Solar Tech. Co., Ltd., China). Also, a light-scattering layer with about 4 μm thickness was applied on top of the transparent layer by using commercial 200 nm TiO_2_ pastes (Dalian HepatChroma Solar Tech. Co., Ltd., China). Then, the TiO_2_ photoanodes were sintered at 500 °C for 1 h before being treated with 0.04 M titanium tetrachloride (TiCl_4_) aqueous solution at 70 °C for 1 h. Finally, the TiO_2_ photoanodes were annealed again at 500 °C for 1 h. The TiO_2_ films were sensitized by immersing in 0.50 mM solution of N719 dye in ethanol for 12 h. The DSSC was assembled into a sandwich structure with the dye-sensitized TiO_2_ electrodes, the electrolyte and a Pt sputtered conducting glass. In addition, a drop of the electrolyte solution (10 mM of LiI, 1 mM of I_2_, and 0.1 mM of LiClO_4_ in acetonitrile) was injected into the clamped electrodes. The active area of the test devices was limited to 0.25 cm^2^. A total of ten cells for each investigated CE were fabricated to obtain a representative result.

### 2.4. Instruments and characterizations

X-ray diffraction (XRD) measurements were performed on an X-ray powder diffractometer (Smartlab-9 X-ray diffractometer, Rigaku, Japan) at 40 kV and 40 mA with Cu Kα (*λ* = 1.5418 Å) in the range of 10–80° (2*θ*). Scanning electron microscopy (SEM) was carried out with a Zeiss Supra 35VP instrument coupled with energy-dispersive X-ray spectroscopy (EDS) operating at 10 kV. X-ray photoelectron spectroscopy (XPS) was performed on ESCALAB 250XI (Thermo Fisher Scientific, America) using Mg Kα radiation as the excitation source.

Electrochemical analyses, including cyclic voltammetry (CV) measurements, Tafel polarization curves and electrochemical impedance spectroscopy (EIS), were conducted on an electrochemical workstation (CHI660E, Shanghai Chenhua Device Company, China). Cyclic voltammetry measurements were performed in a three-electrode system in an acetonitrile solution of 1 mM I_2_, 10 mM LiI and 100 mM LiClO_4_, and the potential range was set from −0.6 V to 1.2 V at a scan rate of 50 mV s^−1^. The FTO glass coated with the catalyst was used as the working electrode; a Pt foil and an Ag/AgCl electrode were used as the counter electrode and the reference electrode, respectively. Tafel polarization curves and electrochemical impedance spectroscopy results were obtained using a symmetrical cell consisting of two same NiCoSe_2_ alloy electrodes (CE/electrolyte/CE). EIS was performed in the frequency range from 100 kHz to 100 mHz with 5 mV amplitude. Tafel polarization curves were scanned from −1.0 to 1.0 V with a scan rate of 10 mV s^−1^. Moreover, the resultant impedance spectra were analyzed by the Z-view software. The incident photon conversion efficiencies (IPCE) were obtained using a Keithley 2000 SourceMeter under the irradiation of a 150 W xenon lamp (Oriel) fitted with a monochromator (Cornerstone 74004) as the monochromatic light source. The photocurrent–voltage (*J*–*V*) characteristics of DSSCs based on the as-prepared CEs and commercial Pt/FTO CE were conducted by a standard solar simulator (Xe Lamp Oriel Sol^3^A™ Class AAA Solar Simulators 94023A, USA) under irradiation of simulated solar light at ambient atmosphere. The *J*–*V* curves were also recorded by the CHI660E electrochemical workstation.

## 3. Results and discussion

### 3.1. Morphology and composition

**Fig. 1:**
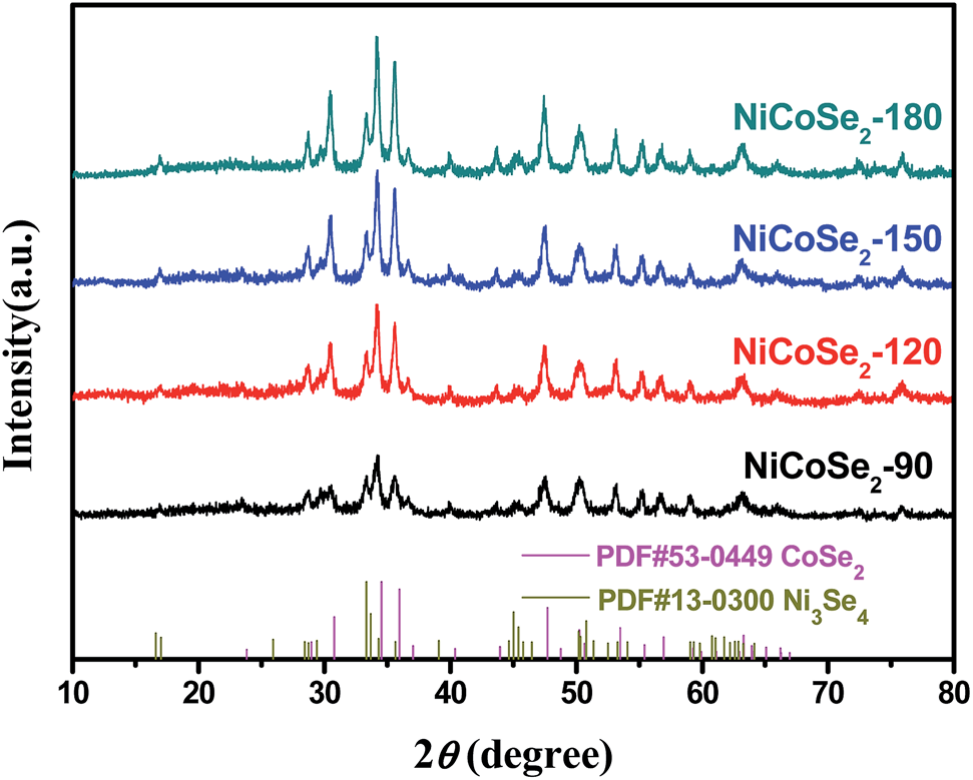
XRD patterns of the samples NiCoSe_2_-90, NiCoSe_2_-120, NiCoSe_2_-150 and NiCoSe_2_-180.

The XRD patterns of NiCoSe_2_ alloys with different hydrothermal temperatures are shown in Fig. 1. It is clearly seen that the four samples show similar XRD patterns except for the NiCoSe_2_-90 sample, which exhibits low crystallinity. The diffraction peaks of NiCoSe_2_-90, NiCoSe_2_-120, and NiCoSe_2_-150 can be readily indexed to orthorhombic CoSe_2_ (JCPDS card no. 53-0449)^21^ and a small fraction of monoclinic Ni_3_Se_4_ (JCPDS card no. 13-0300).^7^ It is clear that there are no other peaks in the patterns, which indicates that the prepared products are of pure quality. Several well-resolved peaks appear at 23.8°, 28.7°, 30.5°, 34.2°, 35.6°, 37.0°, 40.1°, 43.6°, 47.5°, 50.2°, 53.2° and 63.1° corresponding to orthorhombic CoSe_2_ (110), (011), (101), (111), (120), (200), (210), (121), (211) (002), (031) and (122). These peaks also match well with the reported data.^24^ The peaks at around 16.9°, 33.4°, 45.4°, 50.2° and 53.2° corresponding to the (002) (202), (204) (020) and (002) crystal planes of monoclinic Ni_3_Se_4_ are observed. The XRD patterns of the as-prepared NiCoSe_2_ powders mainly consist of two phases, *i.e.*, orthorhombic CoSe_2_ and a small amount of monoclinic Ni_3_Se_4_. Hence, NiCoSe_2_ alloys with nearly same crystal structures have been successfully prepared. In addition, the peak intensity increases with the increasing hydrothermal temperature, indicating improved crystallization and particle size growth; these results will be confirmed by SEM results shown later.

**Fig. 2:**
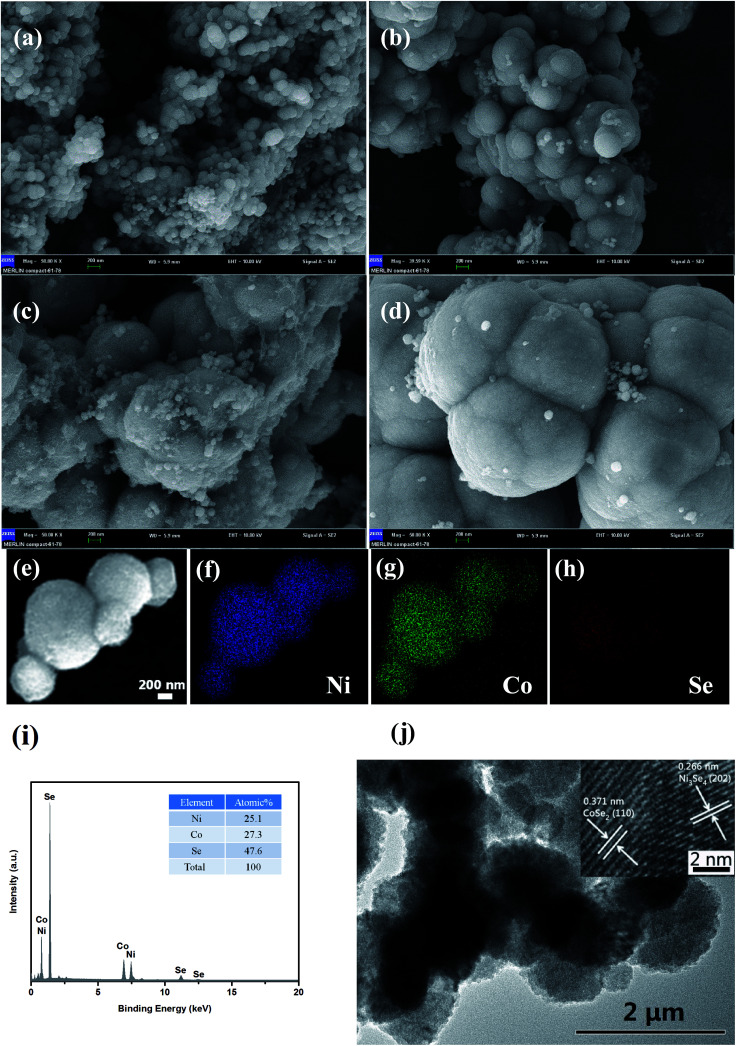
SEM images (scale bar = 200 nm) of NiCoSe_2_ prepared at different reaction temperatures of (a) 90 °C (b) 120 °C, (c) 150 °C and (d) 180 °C. Elemental mapping images (e–h), EDS (i), TEM image (j) and HRTEM (inset of (j)) of NiCoSe_2_-150.

SEM images were obtained for the investigation of the structural morphologies of the synthesized samples. As shown in Fig. 2a, NiCoSe_2_-90 was composed of a large number of dense spherical particles with a diameter of about 200 nm. When the hydrothermal temperature was increased to 150 °C, the size of NiCoSe_2_ spheres became larger; this was consistent with the XRD analysis. The SEM image of NiCoSe_2_-150 (Fig. 2c) revealed that NiCoSe_2_ spheres with a diameter of about 800 nm were obtained as some of the spheres were partially broken, which can be clearly observed from the flocculent structure of the as-synthesized NiCoSe_2_ spheres. Moreover, the unique flocculent structure of the NiCoSe_2_-150 sample could provide effective transport pathways for electrons and ions, which enhanced the electrochemical kinetics. However, NiCoSe_2_-180 (Fig. 2d) displayed a cauliflower-like aggregated structure since the spheres were connected to each other, and it was difficult to separate out a single sphere, which might have been affected by the relatively high temperature of the hydrothermal process. The atomic ratios of the prepared Ni–Co–Se alloys were determined by EDS using NiCoSe_2_-150 as a representative sample. The elemental concentrations of Ni, Co and Se in the film were ascertained as 25.1, 27.3 and 47.6, respectively. This ratio was close to the composition of NiCoSe_2_ (1 : 1 : 2). Furthermore, as can be seen in Fig. 2e–h, Ni, Co and Se elements displayed a uniform distribution in the mapping diagram, which further confirmed that the Ni–Co–Se alloys have been successfully synthesized. The TEM image (Fig. 2j) further confirmed the sphere size and the overall morphology of NiCoSe_2_-150. The typical HRTEM image (the inset of Fig. 2j) revealed clear lattice fringes separated by 0.371 nm and 0.266 nm, corresponding to the (110) plane of orthorhombic CoSe_2_ and the (202) plane of monoclinic Ni_3_Se_4_, which agreed well with the XRD results.

**Fig. 3:**
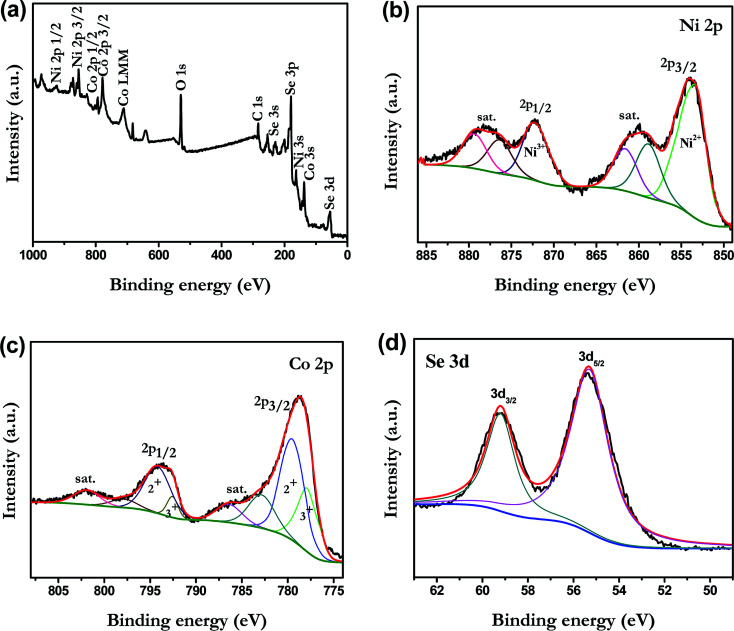
XPS spectra of NiCoSe_2_-150: (a) survey spectrum, (b) Ni 2p, (c) Co 2p and (d) Se 3d.

To investigate the chemical bonding states and the elemental components of the NiCoSe_2_ alloy, the sample was examined by X-ray photoelectron spectroscopy (XPS). The NiCoSe_2_-150 sample was selected as a representative sample. As shown in Fig. 3a, the survey spectrum confirmed the presence of C, O, Ni, Co and Se in the product. The C 1s peak and the O 1s peak appeared because the surface was exposed in ambient conditions, and it adsorbed other elements inevitably, which indicated that the near-surface of the NiCoSe_2_-150 sample mainly comprised Se, Ni, and Co.^25^Fig. 3b–d show the high-solution XPS spectra of Ni 2p, Co 2p and Se 3d, respectively. The high-solution spectra of Ni 2p and Co 2p could be divided into spin–orbit doublets and shakeup satellites (identified as “Sat.”) by the Gaussian fitting method. The Ni 2p spectrum of NiCoSe_2_-150 is shown in Fig. 3b; the fitting peak at 853.3 was assigned to Ni^2+^, and the peak at 872.5 eV was ascribed to Ni^3+^.^26,27^ For the Co spectrum, the first doublet (at 777.9 and 792.6 eV) and the second doublet (at 779.7 and 795.1 eV) were the characteristics of Co^3+^ and Co^2+^.^25,28^ Our results showed that the Co and Ni atoms were in divalent states. Moreover, as shown in Fig. 3d, Se 3d peaks could be deconvoluted into two peaks; the two peaks at 59.2 eV and 55.3 eV could be assigned to Se 3d_3/2_ and 3d_5/2_, respectively, which represented typical metal–selenium bonds.^29^ According to the XPS analysis, it was clear that the surface of the Ni–Co–Se alloy mainly consisted of Ni^2+^, Co^2+^ and Se^2−^.

### 3.2. Electrochemical properties

**Fig. 4:**
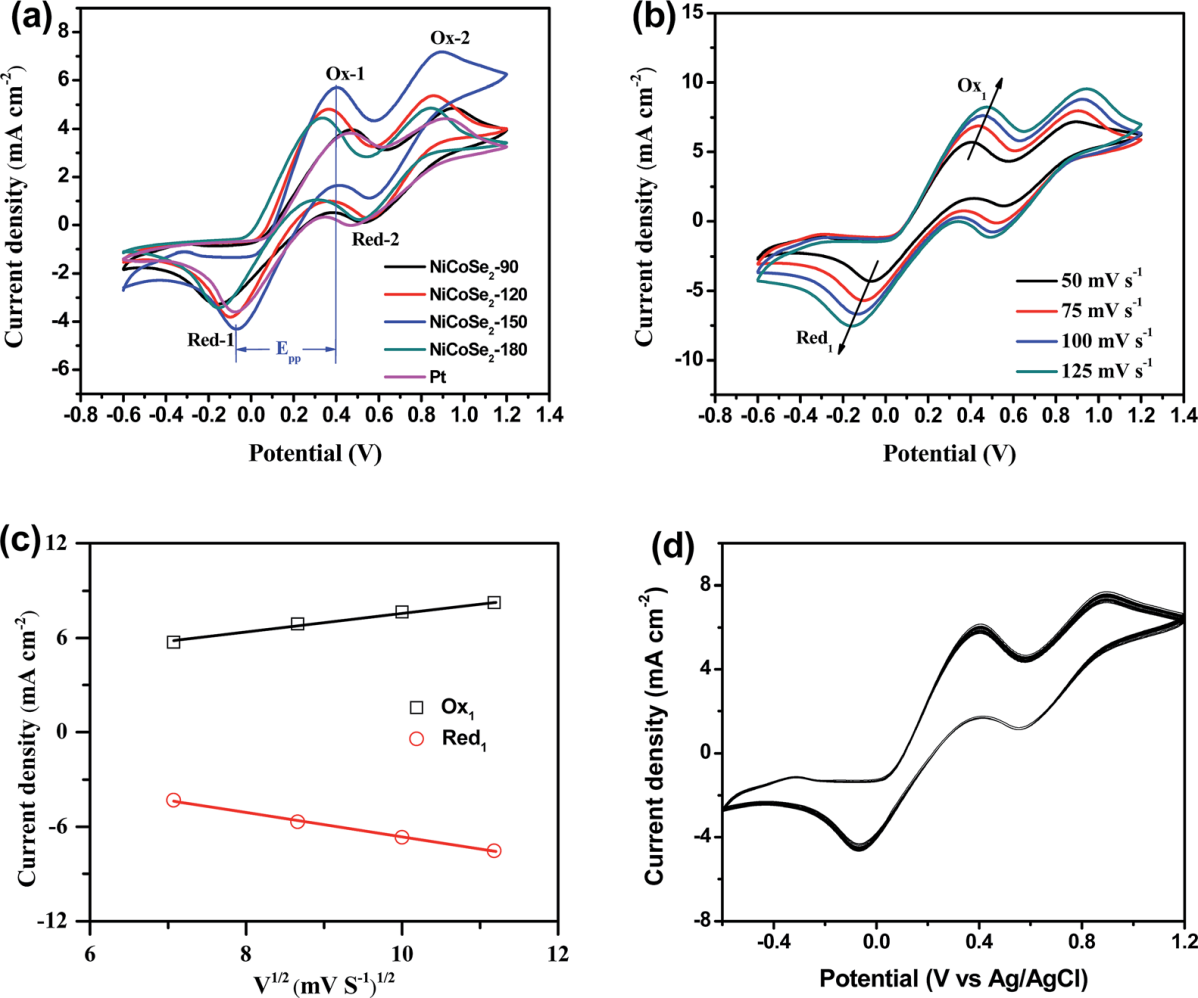
(a) CV curves of NiCoSe_2_-90, NiCoSe_2_-120, NiCoSe_2_-150, NiCoSe_2_-180 and Pt CEs for I^−^/I_3_^−^ redox couples at a scan rate of 50 mV s^−1^, (b) CVs for the NiCoSe_2_-150 electrode recorded at different scan rates of 50, 75, 100 and 125 mV s^−1^ and (c) the relationship between the redox current density and the square root of scan rates of CVs for NiCoSe_2_-150 CE. (d) The 100-stacking CV curves from NiCoSe_2_-150 CE at a scan rate of 50 mV s^−1^.

To evaluate the electrocatalytic properties of the as-prepared NiCoSe_2_ microspheres, cyclic voltammetry (CV) and electrochemical impedance spectroscopy (EIS) were carried out. CV was performed to evaluate the reaction kinetics of the I^−^/I_3_^−^ redox reaction.^30^ As shown in Fig. 4a, all CEs showed two pairs of redox peaks (Red-1/Ox-1, Red-2/Ox-2), of which the redox peaks at the more negative potentials represent the eqn (1) (Red-1/Ox-1) reaction, and the peaks at the more positive potentials represent the eqn (2) (Red-2/Ox-2) reaction.

1 3I_2_ + 2e^−^ ↔ 3I^−^

2 I_3_^−^ + 2e^−^ ↔ 3I^−^

The I_3_^−^ reduction reaction mainly occurs on the side of CE; thus, the I^−^/I_3_^−^ redox couple was explored in this study. Generally, the peak-to-peak potential separation (*E*_pp_) between Red-1 and Ox-1 and the peak current density of the reduction peak Red-1 (|*J*_Red-1_|) are two crucial parameters in a CV curve. Moreover, the value of |*J*_Red-1_| is always utilized to assess the reaction rate of the CE catalyst for the reduction reaction of I_3_^−^ to I^−^. A higher peak current density |*J*_Red-1_| results in enhanced electrocatalytic activity of the CE material. As shown in Table 1, NiCoSe_2_-150 CE possessed the highest |*J*_Red-1_| value of 4.31 mA cm^−2^, suggesting that this sample has higher I^−^/I_3_^−^ redox couple electrocatalytic ability compared to Pt (3.59 mA cm^−2^). Moreover, the |*J*_Red-1_| value of NiCoSe_2_-120 (3.81 mA cm^−2^) was slightly larger than that of Pt CE. On the other hand, *E*_pp_ is a parameter to evaluate the reversibility of the redox reaction, and a lower *E*_pp_ value means better electrocatalytic ability. The *E*_pp_ values of CEs increased in the order NiCoSe_2_-150 (461 mV) < NiCoSe_2_-120 (465 mV) < NiCoSe_2_-180 (487 mV) < Pt (535 mV) < NiCoSe_2_-90 (633 mV). Apparently, NiCoSe_2_-120 and NiCoSe_2_-150 CEs showed significantly higher electrocatalytic activities than Pt. Additionally, the highest |*J*_Red-1_| and lowest *E*_pp_ values indicated that NiCoSe_2_-150 CE has superior electrocatalytic activity for I_3_^−^ reduction, due to which it can be a better alternative material to Pt CE in DSSCs.

**Table 1:** Electrochemical parameters obtained from CV, EIS and Tafel characterizations

CEs	*E*_pp_ (mV)	|*J*_Red-1_| (mA cm^−2^)	*R*_s_ (Ω cm^2^)	*R*_ct_ (Ω cm^2^)	*J*_0_ (mA cm^−2^)	*J*_lim_ (mA cm^−2^)
NiCoSe_2_-90	633	3.28	9.4	12.5	1.28	33.73
NiCoSe_2_-120	465	3.81	9.6	3.1	6.53	84.72
NiCoSe_2_-150	461	4.31	9.6	1.8	10.67	118.85
NiCoSe_2_-180	487	3.43	9.5	2.5	7.41	99.54
Pt	535	3.59	9.5	6.2	5.46	79.07

In addition, Fig. 4b shows CVs of the I^−^/I_3_^−^ system on the NiCoSe_2_-150 electrode with different scan rates (*i.e.*, 50, 75, 100 and 125 mV s^−1^). With the increasing scan rate, CVs exhibit a regularly outward extension of all peaks. Fig. 4c illustrates the linear relationship between the peak current density and the square root of the scan rate, indicating that the diffusion of I^−^ controls the redox reaction on CE and there is no specific interaction between the prepared CE and the I^−^/I_3_^−^ redox pair.^31^ To investigate the stability of CE in the liquid electrolyte, CV measurements have been performed on the CE based on NiCoSe_2_-150 at a scan rate of 50 mV s^−1^ for 100 cycles (see Fig. 4d). No apparent decrease in the current density is observed during cycling, indicating that this CE exhibits good electrochemical stability as the CE material for DSSCs.

**Fig. 5:**
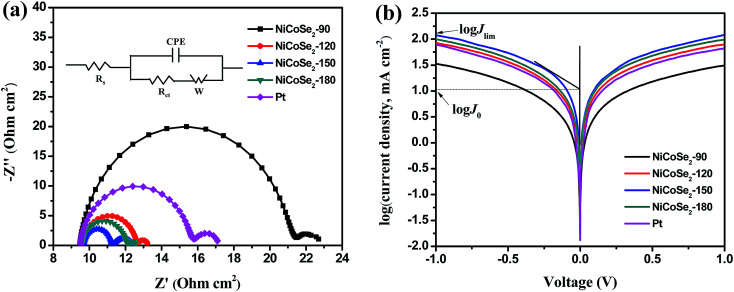
(a) EIS Nyquist plots and the corresponding equivalent circuit model (inset) and (b) Tafel polarization curves for the symmetric cells fabricated with NiCoSe_2_-90, NiCoSe_2_-120, NiCoSe_2_-150, NiCoSe_2_-180 and Pt electrodes.

EIS analysis was conducted using typical symmetrical dummy cells for further characterization of the charge transfer process at the electrolyte/CE interface. Nyquist plots of all cells with Ni–Co–Se alloy CEs (Fig. 5a) showed two semicircles at the low-frequency and the high-frequency regions. The intercept of the high-frequency semicircle with the horizontal axis signifies the series resistance (*R*_s_), which can be affected by the adhesion of the electrocatalyst film to the FTO glass. The high-frequency semicircle corresponds to the charge transfer resistance (*R*_ct_) at the CE/electrolyte interface for the I_3_^−^ reduction, which is a pivotal parameter reflecting the catalytic activity of the prepared CEs. A smaller value of *R*_ct_ stands for a smaller value of the overpotential needed for the electron transfer from CE to electrolyte.^32,33^ The low-frequency semicircle (right-hand side) represents the Warburg diffusion resistance (ZW) within the bulk electrolyte and the electrocatalyst film. The above-mentioned parameters were extracted by fitting the EIS spectrum with an equivalent circuit (the inset of Fig. 5a), and the results are shown in Table 1. As shown in Table 1, the *R*_s_ values of NiCoSe_2_-90, NiCoSe_2_-120, NiCoSe_2_-150, NiCoSe_2_-180 and Pt were 9.4, 9.6, 9.6, 9.5 and 9.5 Ω cm^2^, respectively. A larger *R*_s_ value indicates weaker adhesion of the electrocatalytic material to the FTO substrate. All *R*_s_ values were very close to each other. In addition, *R*_ct_ increased in the order NiCoSe_2_-150 (1.8 Ω cm^2^) < NiCoSe_2_-180 (2.5 Ω cm^2^) < NiCoSe_2_-120 (3.1 Ω cm^2^) < Pt (6.2 Ω cm^2^) < NiCoSe_2_-90 (12.5 Ω cm^2^), which indicated that the *R*_ct_ values of NiCoSe_2_-120, NiCoSe_2_-150 and NiCoSe_2_-180 were all smaller than that of Pt. It is thus apparent that the above-mentioned three devices have higher catalytic activities and charge-transfer abilities than the Pt-based DSSC.

Tafel polarization analysis was carried out to estimate the electrocatalytic activity of the charge-transfer performance of the I^−^/I_3_^−^ redox pair at the CE/electrolyte interface. Typically, a Tafel curve can be divided into three zones by the value of the overpotential. The region at the lower overpotential of |*U*| < 0.120 V represents the polarization zone, the region at the middle potential with a sharp slope represents the Tafel zone, and the region at higher overpotential with the horizontal portion represents the diffusion zone. The exchange current density (*J*_0_) is estimated from the slope of the cathodic or the anodic branch, and the limiting diffusion current density (*J*_lim_) is the intersection of the anodic branch with the *Y*-axis. *J*_0_ and *J*_lim_ can be obtained from the corresponding Tafel zone and the diffusion zone.^34–37^ As shown in Fig. 5b, NiCoSe_2_-150 CE achieved the highest *J*_0_ value (10.67 mA cm^−2^) in the Tafel zone, and it exhibited the strongest catalytic activity. The *J*_0_ values for these electrodes followed the order NiCoSe_2_-150 > NiCoSe_2_-180 > NiCoSe_2_-120 > Pt > NiCoSe_2_-90, suggesting that the catalytic activities of CEs could be presented in the same order. In the diffusion region, NiCoSe_2_-120, NiCoSe_2_-150 and NiCoSe_2_-180 CEs exhibited higher *J*_lim_ values compared to Pt, which indicated that these prepared electrodes can achieve higher catalytic activities. NiCoSe_2_-90 CE showed the lowest *J*_lim_ value, which suggested the lowest electrocatalytic ability towards I_3_^−^ reduction. The trend of *J*_lim_ was consistent with that of *J*_0_ for these CEs. Additionally, the *J*_0_ values could be calculated by using eqn (3), and the parameters obtained from the Tafel plots are given in Table 1. Larger *J*_0_ values result in smaller *R*_ct_ values of CEs, which was in agreement with the EIS data. Clearly, the above-mentioned CV, EIS, and Tafel polarization measurement results indicated that the NiCoSe_2_-150 electrode had the highest catalytic activity as a CE compared to the other NiCoSe_2_ CEs and Pt CE. In contrast, NiCoSe_2_-120, NiCoSe_2_-150, NiCoSe_2_-180 showed higher catalytic activities than Pt and exhibited potentials to achieve reasonably high PCE values.

3 *J*_0_ = *RT*/*nFR*_ct_

here, *R*_ct_ is the charge transfer resistance at the CE/electrolyte interface obtained from the EIS spectra, *F* is the Faraday's constant, *n* is the number of electrons involved in the reduction of I_3_^−^ at the electrode (*n* = 2), *T* is the temperature (298 K), and *R* is the gas constant.

### 3.3. Photovoltaic performances

**Fig. 6:**
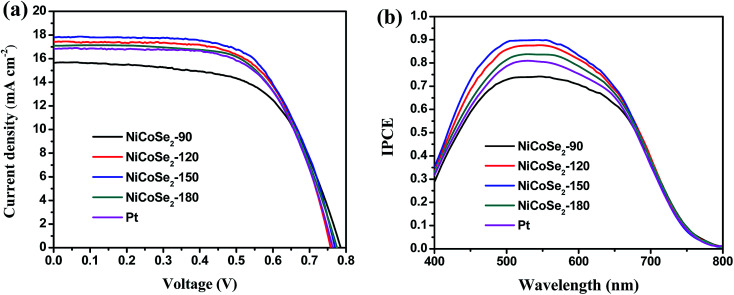
(a) *J*–*V* curves (b) IPCE curves of DSSCs with NiCoSe_2_-90, NiCoSe_2_-120, NiCoSe_2_-150, NiCoSe_2_-180 and Pt-based CEs.

Fig. 6a shows the *J*–*V* curves of DSSCs with NiCoSe_2_ CEs and Pt CE, and the corresponding photovoltaic parameters (including the open circuit voltage (*V*_oc_), short circuit current density (*J*_sc_), fill factor (FF) and PCE) are presented in Table 2. PCE increased in the order NiCoSe_2_-90 (7.56%) < Pt (8.22%) < NiCoSe_2_-180 (8.31%) < NiCoSe_2_-120 (8.48%) < NiCoSe_2_-150 (8.76%), which showed that NiCoSe_2_ alloy CEs exhibited excellent electrocatalytic activities for the reduction of I_3_^−^. DSSCs based on NiCoSe_2_-150 CE showed the highest PCE of 8.76%, the highest *J*_sc_ of 17.82 mA cm^−2^, *V*_oc_ of 0.770 V and FF of 0.638. DSSCs with NiCoSe_2_-90 CE showed distinct lowest PCE and a *J*_sc_ value of 15.65 mA cm^−2^. The DSSC with NiCoSe_2_-120 exhibited higher *J*_sc_ and PCE values compared to NiCoSe_2_-90. In addition, the DSSC with NiCoSe_2_-180 CE also exhibited remarkable electrocatalytic activity with high PCE and a *J*_sc_ value of 17.08 mA cm^−2^; these values were slightly higher than those of Pt.

**Table 2:** Photovoltaic data of DSSCs with NiCoSe_2_-90, NiCoSe_2_-120, NiCoSe_2_-150, NiCoSe_2_-180 and Pt-based CEs

CEs	*V*_oc_ (V)	*J*_sc_ (mA cm^−2^)	FF	PCE (%)
NiCoSe_2_-90	0.786 ± 0.014	15.65 ± 0.011	0.615 ± 0.010	7.56 ± 0.25
NiCoSe_2_-120	0.760 ± 0.012	17.44 ± 0.011	0.640 ± 0.011	8.48 ± 0.24
NiCoSe_2_-150	0.770 ± 0.008	17.82 ± 0.007	0.638 ± 0.009	8.76 ± 0.22
NiCoSe_2_-180	0.774 ± 0.012	17.08 ± 0.011	0.628 ± 0.010	8.31 ± 0.26
Pt	0.765 ± 0.010	16.84 ± 0.008	0.637 ± 0.009	8.22 ± 0.21

To analyse the differences between the *J*_sc_ values of these devices, the IPCE responses as a function of the incident wavelength are plotted in Fig. 6b. The IPCE plot of the DSSC based on NiCoSe_2_-150 covers a broad wavelength range from 400 nm to 800 nm with an IPCE maximum value of 89.9% at 530 nm, and this value matches well with the absorption result of the commercial N719 dye. Moreover, the order of the IPCE values for these devices is NiCoSe_2_-150 > NiCoSe_2_-120 > NiCoSe_2_-180 > Pt > NiCoSe_2_-90, which agrees well with the *J*_sc_ order. With the increase in the hydrothermal temperature, PCEs of NiCoSe_2_-based DSSCs increase first and then decrease with a maximum value of 8.76%. The high IPCE value and the broader absorption of NiCoSe_2_-150 explain the high *J*_sc_ value from the *J*–*V* measurement. The slightly higher efficiency observed for NiCoSe_2_-150 is mainly due to relatively larger *J*_sc_ value, which can be due to the efficient reduction of I_3_^−^ and the reduced charge transfer resistance at the CE/electrolyte interface. NiCoSe_2_-150 CE exhibits superior morphology, which is beneficial to enhance its catalytic activity.

## 4. Conclusions

In conclusion, ternary NiCoSe_2_ alloy-based shape-controllable microspheres with a uniform size were synthesized *via* a simple hydrothermal method. Tuning the hydrothermal temperature could program the shape and the size of NiCoSe_2_ microspheres. The size of the resultant NiCoSe_2_ microspheres increased with the increasing temperature. Also, the interior of NiCoSe_2_-150 possessed a flocculent structure. Subsequently, the synthesized NiCoSe_2_ microspheres were deposited on FTO glass to prepare low-cost and high-performance CEs for DSSCs. The DSSCs based on NiCoSe_2_-120 and NiCoSe_2_-150 CEs exhibited PCEs of 8.48% and 8.76%, respectively, which were higher than that of the DSSC with Pt CE (8.31%). The CV and EIS tests indicated that NiCoSe_2_-150 CE achieved the highest catalytic activity and the lowest charge transfer resistance at the CE/electrolyte interface for the reduction of I_3_^−^/I^−^ compared to the other NiCoSe_2_ CEs and Pt CE; these results agreed with the results of the photovoltaic analysis. This research indicates that NiCoSe_2_ microspheres can be used as low-cost and highly efficient CEs in DSSCs.

## Conflicts of interest

The authors declare no conflict of interest.

## Supplementary Material
